# Night workers have lower levels of antioxidant defenses and higher levels of oxidative stress damage when compared to day workers

**DOI:** 10.1038/s41598-019-40989-6

**Published:** 2019-03-14

**Authors:** Kely R. C. Teixeira, Camila P. dos Santos, Luciana A. de Medeiros, Jordane A. Mendes, Thúlio M. Cunha, Kátia De Angelis, Nilson Penha-Silva, Erick P. de Oliveira, Cibele A. Crispim

**Affiliations:** 1 Faculty of Medicine, Federal University of Uberlândia, Uberlândia, MG Brazil; 2 Department of Physiology, Federal University of São Paulo, São Paulo, SP Brazil; 3 Institute of Biotechnology, Federal University of Uberlândia, Uberlândia, MG Brazil

## Abstract

The effects of circadian misalignment and work shift on oxidative stress profile of shift workers have not been explored in the literature. The present study aimed to evaluate the role of shift work (day and night) and social jetlag - a measure of circadian misalignment - with oxidative stress markers. A cross-sectional study was performed with 79 men (21–65 years old, 27.56 ± 4.0 kg/m^2^) who worked the night shift (n = 37) or daytime (n = 42). The analyzed variables included anthropometric measures and determination of systemic levels of markers of oxidative damage and antioxidant defense. Social jetlag was calculated by the absolute difference between the mean sleep point on working and rest days. The night group presented higher systemic values of thiobarbituric acid reactive substances and hydrogen peroxide, and lower levels of nitrite, total antioxidant capacity, and catalase and superoxide dismutase activities in relation to the day group. However, social jetlag was not associated with oxidative stress-related biomarkers analyzed in the night group. These results suggest that the night worker has higher levels of oxidative stress damage and lower levels of antioxidant defenses, while social jetlag was not a possible responsible factor for this condition.

## Introduction

In Western society, the demand for work by companies and services for 24 hours a day and seven days a week is increasing. As a result, the number of shift workers has increased massively in the last decades, corresponding to about 10% to 20% of the workforce in Europe and the United States^1^ and approximately 14.9% in Brazil^2^. However, this work modality leads to circadian misalignment^3–5^, which is associated with the onset of several pathological conditions such as dyslipidemia^6^, obesity^7^, metabolic syndrome^8^, type 2 diabetes mellitus,^9^ cardiovascular diseases^10^ and cancer^11^.

Currently, circadian misalignment has been measured by the calculation of social jetlag (SJL)^10,12^, a term used in similarity to the jetlag resulting from trans-meridional journeys. However, unlike displacement jetlag, SJL occurs chronically throughout the professional life of the individual and can lead to chronic health effects^13^. Indeed, SJL has been associated with biomarkers of inflammation and diseases such as diabetes and obesity^14^, as well as smoking, alcohol abuse and sedentary lifestyle^15^.

The circadian system represents a complex temporal regulatory network, which plays an important role in the synchronization of various biological processes within the organism and in its coordination with the environment. Circadian disorders, caused by work shift, may lead to the desynchronization of multiple physiological processes and the disruption of normal homeostasis in tissues^16^, and, with that, an increase in inflammatory activity^17^, disturbance in the activity of the neuroendocrine stress system^18,19^, reduction of immunological defenses^19^ and excessive formation of reactive oxygen species (ROS)^19,20^.

ROS is a very broad term that encompasses, in addition to free radicals (hydroxyl radical, nitric oxide and superoxide radical), other non-radical species also derived from oxygen, such as hydrogen peroxide^21,22^. Oxidative stress, a condition that characterizes the imbalance between oxidative and antioxidant compounds, due to the excessive generation of free radicals or a deficiency in the capacity to fight them^23,24^, leads to the oxidation of biomolecules, with consequent loss of their biological functions and generation of homeostatic breaks that can affect the cells, tissues and organs^25,26^. Oxidative stress is considered a cardiometabolic risk factor^27^ and has been related to the pathophysiology of a wide variety of diseases^28,29^, many of them highly frequent in shift workers^30,31^.

Although these concepts are already individually established in the literature^24,32^, there is still limited evidence on the relationship between SJL, oxidative stress, antioxidant defenses and shift work. Given the importance of investigating the connection of oxidative stress to the health-illness relation of shift workers, this study was designed from the hypothesis that night workers present higher values of oxidative stress markers and lower levels of antioxidant defenses in relation to day workers. Also, we posited that SJL is positively associated with oxidative stress markers and negatively associated with antioxidant defenses in these workers. Thus, the objective of the present study was to evaluate the role of the work shift (day and night) and SJL on oxidative stress markers.

## Results

### Participant characteristics

The volunteers were between 21 and 65 years of age. There was no difference between the shifts in relation to the workers’ age (night: 42.43 ± 8.50 years; day: 43.40 ± 12.72 years, *p* = 0.688) and working time in the current shift (Table 1). Of the 37 night workers evaluated, 13 (35%) had daytime shifts, and only 10 (27%) did not take additional shifts. In addition, there was also no difference between the shifts in relation to body weight (night: 83.49 ± 11.73 kg; day: 81.13 ± 13.97 kg, *p* = 0.427), body mass index (BMI; night: 27.24 [26.05–29.64] kg/m²; day: 26.51 [24.00–28.49] kg/m², *p* = 0.115), waist circumference (WC; night: 97.62 ± 11.04 cm; day: 95.17 ± 11.04 cm, *p* = 334) and duration of physical exercise per week (night: 240.0 [157.50–330.00] min; day: 240.0 [135.00–435.00] min, *p* = 0.601). On the other hand, night workers presented higher workload (*p* < 0.001), daytime sleepiness (*p* = 0.002) and SJL (*p* < 0.001), as well as shorter sleep time in workdays (*p* < 0.001) in relation to day workers (Table 1).

**Table 1:** Working hours per week, sleep patterns, score sleepiness, chronotype and social jetlag of employees according to shift worked.

	Night (n = 37)	Day (N = 42)	p-value
**Working Hours/week**	57.0 [42.0–69.0]	36.0 [36.0–40.0]	<0.001*
**Working time** (years)	5.00 [2.00–12.5]	4.00 [2.00–10.75]	0.348
**Sleepiness Score (** ***Epworth*** **)**	10.76 ± 4.88	7.48 ± 4.00	0.002*
Daytime Sleepiness	16 (43.2)	9 (21.4)	0.037*
**No sleepiness**	21 (56.8)	33 (78.6)	
**Mean Sleep Duration (h)**			
Work days	3:50 [2:22–4:27]	6:35 [5:28–7:35]	<0.001*
Rest days	7:56 ± 1:58	8:33 ± 1:52	0.170
**Chronotype (MSF** ^**E**^ **sc) (h)**	3:44 ± 1:00	3:38 ± 1:25	0.708
Morning	21 (56.8)	29 (69.0)	0.260
Indifferent	12 (32.4)	7 (16.7)	
Evening	4 (10.8)	6 (14.3)	
**Social Jetlag (h)**	5:07 [2:35–7:53]	1:15 [0:45–2:02]	<0.001*
Yes	32 (86.5)	25 (59.5)	0.011*
No	5 (13.5)	17 (40.5)	

Values are presented as mean ± SD for normally distributed data or median (interquartile range) for non-normally distributed data. Comparisons between groups were done using the Student’s t-test or the Mann-Whitney test, for independent samples, for data with and without normal distribution, respectively, or by the Chi-square test, for variables expressed as frequency. *p < 0.05 indicates statistically significant difference. SJL was calculated based on the absolute difference between the average sleep time on working and rest days and was dichotomically categorized as >60 min (with SJL) or <60 min (without SJL).

### Comparison of parameters of oxidative stress damage and antioxidant defense between night and day workers

**Figure 1:**
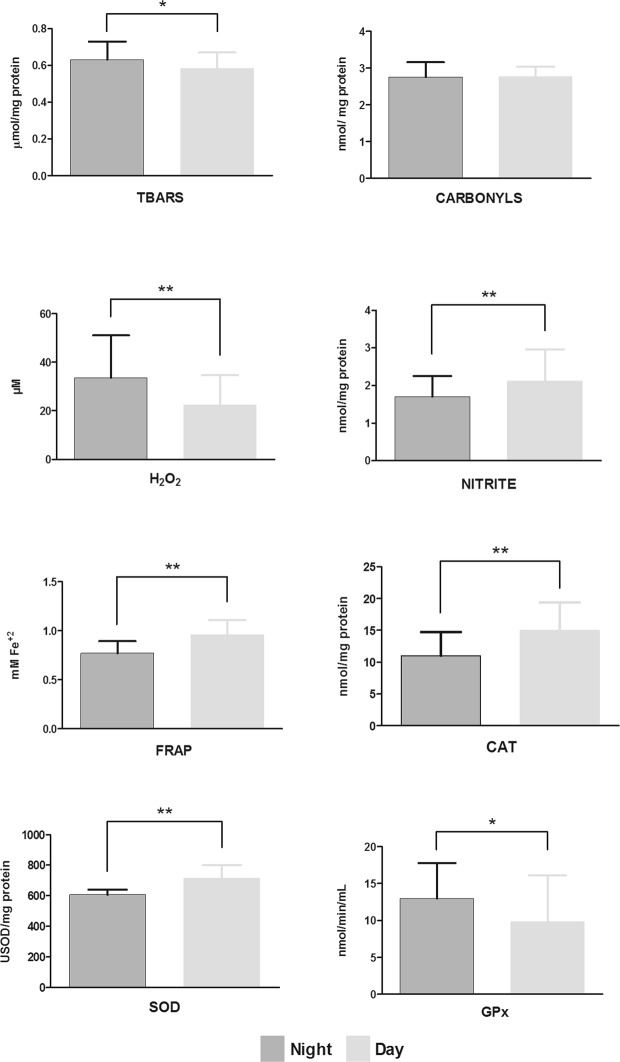
Comparison of parameters of oxidative stress damage (TBARS, thiobarbituric acid reactive substances; carbonyls, plasma protein oxidation); prooxidants (H_2_O_2_, hydrogen peroxide; and nitrite) and antioxidant defense (FRAP, ferric reducing/antioxidant power; CAT, catalase; SOD, superoxide dismutase; and GPx, glutathione peroxidase) between night (dark grey) and day (light grey) workers, using Generalized Linear Model (GzLM) test, adjusted for age and working hours. *p < 0.05 and **p < 0.01 indicates statistically significant difference.

The parameters of oxidative stress between the work shifts are presented in Fig. 1.

Significantly higher values were found in night workers compared to day workers for the variables of lipoperoxidation (thiobarbituric acid reactive substances; TBARS), 0.63 [0.60–0.66] and 0.57 [0.55–0.61] µmol/mg protein (*p* = 0.016); H_2_O_2_, 33.55 [28.17–39.96] and 22.50 [19.11–26.49] µM (*p* = 0.001); and GPx, 12.97 [10.76–15.64] and 9.85 [8.23–11.79] nmol/min/mL (*p* = 0.037), respectively (Fig. 1).

On the other hand, significantly lower values were found in night workers in relation to day workers for the variables nitrite, 1.70 [1.51–1.91] and 2.12 [1.91–2.36] nmol/mg protein (*p* = 0.005); total antioxidant capacity (FRAP), 0.78 [0.73–0.83] and 0.95 [0.89–0.99] mM Fe(ii) (*p* < 0.001); CAT 11.07 [9.77–12.55] and 14.89 [13.27–16.70] nmol/mg protein (*p* = 0.003) and SOD, 609.11 [587.60–631.41] and 713.28 [688.91–738.50] USOD/mg protein (*p* < 0.001), respectively (Fig. 1).

Only protein oxidation values of night and day workers, 2.76 [2.65–2.87] and 2.77 [2.67–2.87] nmol/mg protein, respectively, did not differ significantly (*p* = 0.918).

### Main effects of work shift, SJL and their interaction for parameters of oxidative damage and antioxidant defenses

Table 2 displays an effect of the work shift on the variables FRAP (*p* = 0.002), CAT (*p* = 0.034) and SOD (*p* < 0.001). In this analysis, significantly lower values were found in night workers compared to day workers for the variables FRAP, CAT and SOD, respectively. None of the analyses found interactions between work shift and SJL.

**Table 2:** Main effects of work shift, social jetlag (SJL) and their interaction for parameters of oxidative damage and antioxidant defenses, adjusted for age and working hours.

	Night		Day		Shift		SJL		Shift*SJL	
	With SJL (n = 33)	Without SJL (n = 4)	With SJL (n = 25)	Without SJL (n = 17)	DF	p-value	DF	p-value	DF	p-value
TBARS (µmol/mg protein)	0.62 [0.59–0.66]	0.56 [0.49–0.65]	0.58 [0.54–0.61]	0.61 [0.57–0.66]	1	0.896	1	0.468	1	0.064
Carbonyls (nmol/mg protein)	2.79 [2.66–2.93]	2.51 [2.23–2.81]	2.74 [2.60–2.88]	2.79 [2.62–2.98]	1	0.288	1	0.237	1	0.080
H_2_0_2_ (µM H_2_0_2_)	32.5 [26.2–40.4]	24.5 [14.9–40.3]	26.2 [20.9–32.9]	18.7 [14.2–24.7]	1	0.204	1	0.051	1	0.867
Nitrite (nmol/mg protein)	1.74 [1.53–1.97]	1.64 [1.21–2.21]	2.02 [1.77–2.30]	2.05 [1.75–2.41]	1	0.089	1	0.823	1	0.684
FRAP (mM Fe^+2^)	0.78 [0.73–0.83]	0.77 [0.64–0.91]	0.95 [0.89–1.02]	0.95 [0.88–1.03]	1	0.002*	1	0.961	1	0.994
CAT (nmol/mg protein)	11.1 [9.7–12.6]	11.6 [8.1–16.7]	15.2 [13.2–17.4]	14.1 [11.7–16.8]	1	0.034*	1	0.951	1	0.570
SOD (USOD/mg protein)	610 [587–633]	610 [556–669]	712 [684–742]	697 [660–736]	1	<0.001*	1	0.915	1	0.900
GPx (nmol/min/mL)	12.8 [10.3–16.2]	14.4 [8.32–25.1]	8.4 [6.6–10.8]	11.9 [8.90–16.1]	1	0.085	1	0.126	1	0.571

SJL was calculated based on the absolute difference between the average sleep time on working and rest days and was dichotomically categorized as >60 min (with SJL) or <60 min (without SJL).

Abbreviations: TBARS, thiobarbituric acid reactive substances; carbonyls, plasma protein oxidation; H_2_0_2_, oxygen peroxide; FRAP, *ferric* reducing antioxidant power (total antioxidant capacity); CAT: catalase; SOD, superoxide dismutase; GPx, glutathione peroxidase.

*p < 0.05 indicates statistically significant values. Data were represented as mean and Wald confidence interval (95% CI).

### Associations between social jetlag and parameters of oxidative damage and antioxidant defense

When the variables of all volunteers were evaluated without stratification by work shift, through linear regression analysis, associations were not found. With the stratification of the volunteers per shift, a negative association between SJL and TBARS was found in day workers (β = −0.03, *p* = 0.013). Social jetlag was not associated with others oxidative stress-related biomarkers analyzed in day workers. Additionally, no correlations were obtained between SJL and oxidative stress parameters evaluated in the night group (Table 3).

### Associations between sleep duration, sleep debt and parameters of oxidative damage and antioxidant defense

We performed partial Pearson correlation (adjusted for shift) between sleep duration in work days and stress parameters (TBARS: r = −0.25, *p* = 0.057; carbonyls: r = 0.06, *p* = 0.646; nitrite: r = 0.13, *p* = 0.331; H_2_O_2_: r = 0.13, *p* = 0.331; FRAP: r = 0.20, *p* = 0.380; CAT: r = −0.05, *p* = 0.716; SOD: r = 0.15, *p* = 0.275; GPx: r = −0.08, *p* = 0.543), and mean sleep duration and stress parameters (TBARS: r = −0.18, *p* = 0.179; carbonyls: r = −0.09, *p* = 0.519; nitrite: r = 0.04, *p* = 0.768; H_2_O_2_: r = 0.24,* p* = 0.067; FRAP: r = 0.05, *p *= 0.739; CAT: r = −0.12, *p* = 0.381; SOD: r = 0.17, *p* = 0.191; GPx: r = −0.10, *p* = 0.441) and no association was found. Similarly, we found no association in the partial Pearson correlation (adjusted for shift) between sleep debt and stress parameters (TBARS: r = 0.12, *p* = 0.358; carbonyls: r = −0.16, *p* = 0.243; H_2_0_2_: r = 0.12, *p* = 0.389, nitrite: r = −0.03,* p* = 0.827; FRAP: r = −0.12, *p* = 0.391; CAT: r = −0.02, *p* = 0.901; SOD: r = −0.09, *p* = 0.504; GPx: r = −0.04, *p* = 0.758).

## Discussion

This study aimed to evaluate the oxidative stress profile according to the work shift and the circadian misalignment associated with SJL. The night workers had lower levels of antioxidant defense and higher levels of ROS and oxidative stress damage when compared to day workers, which confirms the initial hypothesis of this study. However, we did not find differences in relation to those variables between individuals with and without SJL or relevant associations of this chronobiological variable and the levels of antioxidant defense enzymes. To the best of our knowledge, this is the first study that showed that the oxidative damage markers suffer a chronic effect of night work.

Some studies have shown a reduction in total antioxidant capacity^18,33^ and an increase in levels of markers of oxidative stress^18^ after continuous hours of shift work. Indeed, the present study found that night workers presented higher values of TBARS and H_2_O_2_ and lower values of nitrite, FRAP, CAT and SOD. Thus, shift work might lead to increased oxidative stress damage (TBARS) in these workers, through an increase in ROS production (H_2_O_2_) and a reduction in enzymatic (CAT and SOD) and non-enzymatic (FRAP) antioxidant defenses. One of the possible explanations for this group’s high oxidative stress profiles may be due the lack of sleep to which these individuals are subjected. Sleep is a dynamic resting state with antioxidant properties, responsible for eliminating the ROS produced during wakefulness^34,35^. Thus, the normal response of oxidative stress could be impaired under conditions of sleep deprivation.

At the same time, the synthesis of adenosine triphosphate (ATP) itself produces ROS, by-products formed as part of the normal aerobic metabolism of mitochondria and peroxisomes, which are neutralized by antioxidant molecules. Therefore, prolonged wakefulness requires higher metabolism to maintain the use of ATP, which necessitates an increased oxygen consumption, resulting in a significantly higher production of oxidants^36^. In addition, the formation of oxidants seems to be under the influence of circadian factors that regulate energetic metabolism^37^. Thus, a circadian misalignment could actually lead to exacerbation of ROS production as a result of changes in mitochondrial functioning^36,38^. In fact, the increase in ROS levels during prolonged wakefulness suggests the existence of an interrelation between redox metabolism and circadian rhythm^35,39^. In our study, however, we found no association between sleep duration, sleep debt and parameters of oxidative damage and antioxidant defense. We believe that the shorter sleep duration of night workers could play a role on oxidative stress parameters, but probably not in a direct and isolated way. In addition to the adaptation of biological rhythms to inversions of periods of rest and activity, these workers are also subject to a drastic change in lifestyle, which negatively influences general health, sleep and social and family interactions^40^. Thus, all these factors can interact and, together, take to higher levels oxidative stress of the volunteers. However, it becomes difficult to isolate a single factor associated with oxidative stress in a real-life study such as ours, given that shift workers are submitted to these different factors together. Randomized clinical trials need to be performed to test whether changes in the sleep duration/sleep debt can influence on oxidative stress in shift workers.

Considering that oxidative stress exerts a harmful effect on vital cell structures^41^, this would be one of the possible mechanisms for the higher incidence of disease in this population. Oxidative damage to DNA, for example, is a factor that has been related to several diseases^42^ and, in the face of oxidative stress, the repair of such damage is essential to prevent such disease onset. Since shift work promotes increased prooxidants but also decreased antioxidant defense - as observed in this study -, the cell is at the mercy of oxidative stress damage.

The decreased antioxidant defense in shift workers appears to occur via suppression of melatonin production^43^, which could - at least in part - justify the results of the present study. Indeed, some studies have shown that a key element in the relationship between work shift and oxidative stress is melatonin^43–45^. Endocrine changes caused by circadian rupture in shift workers result in suppression of melatonin production through artificial light exposure at night, leading to chronically reduced levels of this hormone^46^. Melatonin, exhibits antioxidant properties and is a powerful eliminator of ROS^47^. It is speculated that the increase of oxidative stress (due to constant exposure to artificial light) may be related to both the formation of ROS and the decrease in the secretion of this hormone^48–50^. In this sense, it is possible that it is the low level of circulating melatonin during night work, not adequately compensated for by the sleep period during the day^43^, that explains the association of oxidative stress with the circadian misalignment caused by the night work schedule observed in the present study. In addition, desynchronization of the biological rhythms resulting from shift work leads to deregulation of the hypothalamic-adrenal-pituitary axis, as well as its mediator, cortisol^18^. Such changes in cortisol levels can lead to alterations in the oxidative balance, including alterations in glutathione levels and DNA methylation^51,52^.

It is important to note that SJL reflects the misalignment between the endogenous circadian rhythm and the real time of sleep^32^ and has been associated with several metabolic risk factors, such as alterations in glycemia levels, lipid profile and adiposity^10,13^. In addition, both the expression and activity of antioxidant enzymes and the overall oxidative balance are synchronized with the biological rhythms^53,54^. In the present work, the levels of H_2_O_2_ tend to be higher in individuals with SJL (*p* = 0.051, Table 2), suggesting higher levels of pro oxidant induced by this condition. However, contrary to what we expected, the present study found no significant associations between the SJL and antioxidant defenses analyzing all subjects of this study or the night group. Unexpectedly, we observed a negative and significant association between SJL and TBARS only in day workers (Table 3). This finding is in contrast to the negative physiological effects associated with SJL previously reported in the literature^13–15^. Given the lack of studies that can be compared with our results, as well as the fact that the association between SJL and TBARS is unlike the others found in our study, we suspect that such a finding cannot be used to infer a causal relationship between the variables. In any regard, more studies should be conducted to confirm or discard this relationship found.

**Table 3:** Linear regression of SJL in relation to parameters of oxidative damage and antioxidant defense, adjusted for age, working hours and shift.

	All^a^			Night^b^			Day^b^		
	β	p-value	R^2^	β	p-value	R^2^	β	p-value	R^2^
TBARS	−0.01	0.360	0.13	0.01	0.919	0.01	−0.03	0.013*	0.16
Carbonyls	0.01	0.877	0.01	−0.01	0.983	0.07	−0.05	0.380	0.03
H_2_0_2_	0.62	0.394	0.15	0.28	0.775	0.07	2.56	0.185	0.07
Nitrite	0.01	0.953	0.25	−0.01	0.958	0.08	0.02	0.898	0.20
FRAP	0.01	0.901	0.32	−0.01	0.863	0.08	0.02	0.578	0.20
CAT	−0.16	0.403	0.21	−0.18	0.331	0.06	0.84	0.269	0.04
SOD	−1.33	0.658	0.40	−0.77	0.638	0.06	−6.51	0.656	0.07
GPx	−0.34	0.186	0.10	−0.36	0.136	0.01	−1.02	0.337	0.03

Abbreviations: SJL, Social Jetlag; TBARS, thiobarbituric acid reactive substances; H_2_0_2_, oxygen peroxide; FRAP, *ferric* reducing antioxidant power (total antioxidant capacity); CAT: catalase; SOD, superoxide dismutase; GPx, glutathione peroxidase. ^a^Adjusted for age and shift. ^b^Adjusted for age and working hours.

*p < 0.05 indicates statistically significant adjustments.

In fact, the findings of the present study confirm that work shift can influence the correct functioning of the antioxidant defense mechanisms^55,56^. It is noteworthy that SOD is an enzyme that catalyzes the formation of H_2_O_2_ from the superoxide radical. H_2_O_2_ is responsible for the formation of the hydroxyl radical, but can be removed by a reaction catalyzed by GPx or CAT^19^. GPx has a higher affinity for H_2_O_2_ than CAT, which means that, at low concentrations of H_2_O_2_, GPx plays a much more active role in its removal^57^. In this sense, it is possible that the increased GPx values in the night group may represent an unsuccessful attempt by the organism to adapt to the increased oxidative stress. Moreover, considering that CAT and GPx compete for the same substrate (H_2_O_2_), the responses of these enzymes to increased ROS could be opposite from those previously reported^58^. Furthermore, a loss in antioxidant protection, as verified in the present study, can generate lesions in the cells that would then be repaired with metabolic and molecular adaptive changes that, if not properly controlled, could result in apoptosis and even cell death^38^.

Ulas *et al*.^28^ found that the increase in oxidative stress parameters observed at the end of the night shift in health professionals was not influenced by the position or function at work, but rather by prolonged work activity and inadequate rest time. In the present study, the analyses in Fig. 1 and Table 2 were adjusted for working hours; even so, the differences of these parameters between the shifts remained. Moreover, we performed a partial correlation, adjusted for shift, between working time per week and stress parameters, and found no association with any of the variables evaluated (TBARS: r = 0.06, *p* = 0.651; carbonyls: r = −0.04, *p* = 0.780; H_2_O_2_ values: H_2_O_2_ : r = 0.13; p = 0.331, nitrite: r = −0.17, *p* = 0.206; FRAP: r = 0.04, *p* = 0.788; CAT: r = 0.02, *p* = 0.892; SOD: r = 0.01, *p* = 0.989; GPx: r = −0.02, *p* = 0.403; data not shown in Results section). Thus, it seems that night work promote oxidative stress, mainly affecting antioxidant defenses. It is important to mention that the studies found in the existing literature^18,28,33,59^ evaluated workers at different moments of the work shift (before and after), capturing only the acute effects of the same, instead of their long-term consequences. In the present study, shift workers were compared in a real-life situation, which expresses the chronic effects of shift and desynchronization of biological rhythms in the parameters of oxidative stress damage and antioxidant defense.

Another finding of this study was that night workers had higher SJL, lower average sleep time on workdays and greater daytime sleepiness, compared to daytime workers. In an intervention study, Vetter *et al*.^5^ adjusted the daytime preference of the volunteers to their work shift and found a mean reduction in SJL of 1 h 20 min (*p* = 0.002) and improvement in duration and quality of sleep. For these authors, SJL was found to be lower in individuals whose chronotype was in line with their working hours. Juda *et al*.^60^ showed that SJL depends on the work shift (p < 0.001). As we observed in the current study, these authors^60^ found higher SJL in night shift workers (*p* < 0.05). The results of the present study and others previously found in the literature^5,15,60,61^ clearly indicate the importance of aligning the individual’s chronotype to his or her work shift to minimize the differences between biological and social clocks. This can reduce SJL and, possibly, the consequences of circadian misalignment. However, such alignment is not always possible in real life conditions. In our study, for example, few individuals have an evening chronotype, which brings great difficulty to the decision of how to align chronotypes with the best or preferred shift for work.

This study presents some limitations, such as the variability in the workload among volunteers of both work shifts. For this reason, we adjusted the statistical analyses by working time per week to minimize any effect of this confounding factor. In addition, although the cross-sectional nature of this study does not allow the establishment of causal relationships, the large number of parameters analyzed allowed a better understanding of the oxidative profile in shift workers; this should serve as a basis for future research. We also emphasize, as a limitation, the high variability of sleep cycles in the workers, and the lack of a bona fide marker of circadian rhythmicity. Furthermore, the use of subjective methods those, although widely used in other studies, are dependent on participants’ memory and motivation. In this sense, replacing the questionnaires with objective alternatives - such as actigraphy in the evaluation of the sleep pattern - could provide a more reliable basis. Other limiting factors are the exclusion of female workers, which limits the impact of the current results, and the lack of evaluation of melatonin secretion, which would also help to provide a better understanding of the response of oxidative stress in shift workers. Finally, new studies and new tools will be needed to clarify how the circadian rhythm can modulate the oxidative profile, leading to a new understanding of its outcomes in the health of these workers.

In conclusion, the findings of this study indicate that night workers have lower levels of antioxidant defense and higher levels of ROS and lipoperoxidation, resulting in a condition of oxidative stress that is independent of SJL. More studies are needed to confirm these findings and to define the mechanisms underlying the relationship between shift work and oxidative stress. Preventive changes in working conditions and lifestyle are necessary to improve the health and quality of life of these workers. The effectiveness of these changes could be monitored through the evaluation of oxidative stress status.

## Methods

### Participants and Ethics

This study is in accordance with the Code of Ethics of the World Medical Association (Declaration of Helsinki) and was previously approved by the Human Research Ethics Committee of the Federal University of Uberlândia. The population of this cross-sectional study was composed of male workers from two hospitals in the City of Uberlândia, MG, Brazil, aged between 21 and 65 years. The volunteers worked in the same routine for at least six months in administrative and health-related functions (nurse, physiotherapist, nursing technician, laboratory technician and stretcher bearer) and were classified according to their work shift as: (1) day workers, who worked only during the day, morning and/or afternoon, without developing any work activity at night. If these workers had an extra shift, this occurred only during the day; and (2) night workers, who worked at least six hours after midnight, with and without daytime additional work activities.

One hundred and thirty-five workers were invited to participate in the study. Individuals were excluded from the study if they: were carriers of diseases previously diagnosed and under treatment, except obesity (n = 9); had a different work shift from the two classifications of this study (n = 16) or were smokers (n = 9). In addition, 22 workers refused to participate in the study. Seventy-nine workers (37 night and 42 day workers) remained and completed all data collection. All participants had not recently used any supplements with antioxidant properties. All selected volunteers signed a free and informed consent form to participate in the study.

The volunteers who met the inclusion and exclusion criteria were submitted to anthropometric, chronobiological and sleep evaluations, as well as to blood collection. In addition, data on socio-demographic characteristics, physical exercise and alcohol consumption were also collected. These assessments were made in the morning after a night’s sleep, before the work shift, as oxidative stress parameters do not appear to have important variations in the morning^62^. Individuals were asked to sleep through the whole night (7 to 8 hours) prior to blood collection. At the time of evaluation, blood was only collected if the participants reported that they had followed this guidance. All volunteers were submitted to the same environmental conditions at the time of collection, with artificial lighting and typical hospital noise.

### Anthropometric Assessment

Body mass index (BMI, kg/m²) was calculated from the weight, measured in a scale with 0.1 kg precision (Welmy™, São Paulo, SP, Brazil), and height, measured using a wall-fixed stadiometer with 0.1 cm precision (Welmy™, São Paulo, SP, Brazil)^63^. Waist circumference (WC) was measured between the last costal arch and the iliac crest in volunteers with normal BMI or who were moderately overweight, as recommended by the World Health Organization^63^, and at the navel level in obese volunteers^64^.

### Sleep Pattern, Chronotype and Social Jetlag

These evaluations were performed by a specialized team trained in sleep studies and were based on information compiled in the participants’ responses to a previously reported questionnaire: *What time do you usually go to sleep on work days? How long (how many minutes, on average) do you stay up in bed before falling asleep (after turning off the lights) on work days? At what time do you usually wake up during work days? What time do you usually go to sleep on rest days? How long (how many minutes on average) do you stay up in bed before falling asleep (after turning off the lights) on rest days? What time do you usually wake up on rest days?*^65^. Answers to these questions were used to account for hours of sleep on work and rest days, considering the time it took for each worker to fall asleep (sleep latency).

Sleep duration was calculated using the weighted average of the self-reported sleep duration, given by the following equation: [(sleep duration reported for working days × number of days worked in the week) + (sleep duration reported for rest days × number of rest days in the week)] ÷ 7^66^.

The mid-sleep time on free days (MSF) of the weekend, with a sleep debt correction (MSFsc) given by the difference between the sleep duration on free days and its weekly average, was used to establish the chronotype of the daytime worker^67^. A specific formula proposed by Juda *et al*.^68^ was used to calculate MSF^E^sc and establish the chronotype of the night worker. The shift workers were categorized into early, intermediate and late chronotypes when their MSFsc values were ≤3:59 h, between 4:00 and 4:59 h and ≥5:00 h, respectively^32^. Social Jetlag was estimated with basis in the absolute difference between mid-sleep time on weekends and on weekdays^12^.

### Epworth Sleepiness Scale (ESS)

The Epworth Sleepiness Scale (ESS) is a widely used and reliable predictor of daytime sleepiness^69^, therefore, its Portuguese-language version^70^, previously validated for use by Brazilian participants^71^, was used to evaluate daytime sleepiness. A total score ≥8 was considered indicative of excessive sleepiness^70^.

### Blood Collection and Sample Preparation

Erythrocytes were washed three times with saline and stored in a preservative solution (3.98 mM MgSO4 and 0.96 mM glacial acetic acid) in an ultrafreezer at −80 °C for analysis of the activity of antioxidant defense enzymes.

Blood samples were collected by venipuncture in heparinized tubes (Vacutainer™, BD, Juiz de Fora, MG, Brazil) after 12 h of fasting and immediately centrifuged at 1,300 × *g* for 15 min in a refrigerated centrifuge at 4 °C (Hitachi Koki™, model CFR15XRII Hitachinaka, Japan). The obtained supernatants were then frozen at −80 °C in an ultrafreezer model CUK-UB2I-PW (Panasonic™, Nijverheidsweg, the Netherlands) for further determination of the oxidative stress variables^72^. The erythrocytes were washed three times with saline and stored in a solution of 3.98 mM MgSO4 and 0.96 mM glacial acetic acid in an ultrafreezer at −80 °C for further determination of the activity of antioxidant enzymes. All blood samples were performed in a single moment, the morning after a night’s sleep, before the start of the work shift. The time of blood samples collection varied little between the groups studied: night: 9:00 am [8:00–9:45] and day: 8:00 am [8:00–8:45]; values are expressed in median and interquartile range. This difference occurred due to the fact that we instructed participants to maintain their usual waking hours.

### Determination of Total Plasma Proteins

The protein concentration was determined according to the method previously described by Lowry *et al*.^73^, which uses bovine albumin solution at a concentration of 1 mg/ml as standard and 10 μL of sample.

### Determination of Lipoperoxidation by Dosage of Thiobarbituric-Acid Reactive Substances (TBARS) in Plasma

A volume of 0.75 mL of 10% (w/v) trichloroacetic acid (TCA) was added to 0.25 mL of homogenate to denature its proteins and acidify the reaction medium. This solution was then stirred and centrifuged for three minutes at 1000 rpm. A mixture of 0.5 mL of its supernatant was added to 0.5 mL of 0.67% (w/v) thiobarbituric acid (TBA) was incubated at 100 °C in a thermostated water bath for 15 minutes and then cooled in an ice-water bath. Then, the absorbance generated at 535 nm, due to the reaction of the TBA with lipoperoxidation products present in the biological sample, was measured in a UV-VIS spectrophotometer^74^.

### Determination of Protein Oxidation by Dosage of Carbonyls in Plasma

Oxidatively modified plasma proteins were determined by carbonyls quantification, based on the reaction with 2,4-dinitrophenylhydrazine (DNPH) in 2.5 M HCl, followed by successive washes with organic acids and solvents and final incubation with guanidine (20% v/v TCA; 10% v/v TCA; 1:1 v:v ethanol and ethyl acetate; and 6 M guanidine in 2.5 M HCl pH 2.5)^75^. The absorbance at 360 nm of carbonyls was measured in a UV-VIS spectrophotometer.

### Determination of Superoxide Dismutase (SOD) from Erythrocytes

The determination of the SOD activity was based on the reaction of the superoxide radical with pyrogallol, with the formation of a colored product, detected spectrophotometrically at 420 nm for 2 minutes. The percentage inhibition of initial reaction rates depends on the pH and amount of SOD present in the reaction mixtures. The amount of enzyme required to inhibit the reaction by 50% was defined as one unit of SOD. The reaction mixture contained 980 μL of 50 mM tris-phosphate buffer pH 8.2, 10 μL of 24 mM pyrogallol, 5 μL of 30 mM CAT, and 5 μL of sample. A standard line with three different concentrations of SOD (0.25, 0.5 and 1 U) was obtained to determine the equation used in the calculations^76^.

### Determination of Catalase (CAT) from Erythrocytes

The activity of CAT was determined by measuring the absorbance at 240 nm of a solution of H_2_O_2_. The decomposition rate of hydrogen peroxide is directly proportional to the activity of CAT. Catalase activity was given by peroxide consumption and expressed in nmol/mg protein.

### Determination of Glutathione Peroxidase (GPx) from Erythrocytes

GPx activity was expressed as nmol peroxide/hydroperoxide reduced/min/mg protein and was based on the consumption of NADPH at 480 nm^77^.

### Determination of Total Antioxidant Capacity in Plasma

The total antioxidant capacity of the plasma was determined by the Ferric Reducing Antioxidant Power (FRAP), based on the production of Fe^2+^ (ferrous ion) from the reduction of the Fe^3+^ (ferric ion) present in the 2,4,6-tripyridyl-S-triazine complex (TPTZ). The reduction reaction changes the color of the medium from light purple to dark purple; this allows determination of the total reduction power of a sample by reading the absorbance at 593 nm. The assays were performed on a microplate with the addition of 290 μL of the FRAP reagent (sodium acetate and acetic acid buffer pH 3.6, 10 mM TPTZ and 20 mM ferric chloride hexahydrate) to 10 μL of ferrous sulfate heptahydrate solutions at 0, 0.25, 0.5, and 1 mM (for the standard-line construction) or 10 μL of sample (for FRAP determination by interpolation in the standard line). The microplate was incubated under stirring at 37 °C for 5 min before absorbance readings^78^.

### Determination of Hydrogen Peroxide (H_2_O_2_) in Plasma

The plasma sample was initially incubated for 30 min at 37 °C in 10 mM phosphate buffer containing 140 mM NaCl and 5 mM dextrose. After adding an aliquot of this plasma treated sample to a solution containing 0.28 mM phenol red and 8.5 U/mL horseradish peroxidase (HRPO), and incubating for 5 minutes, a 1 M solution of NaOH was added, before reading of absorbance at 610 nm, which detects the oxidation product formed by the peroxidase-catalyzed reaction of H_2_O_2_ with phenol red. The results were expressed in μM of H_2_O_2_^79^.

### Determination of Total Plasma Nitrite

Total plasma nitrite was determined by reacting the plasma samples with 50 μL of Griess’s reagent and using a standard nitrite curve. Assays were performed on 96-well microplates in an ELISA reader at 592 nm^80^ and values were expressed as nmol/mg protein.

### Statistical Analysis

Initially, the normality of the data was verified with the Shapiro-Wilk Test. Parametric data were presented as means and standard deviations and nonparametric data were presented as median and interquartile range. Comparison of proportions between groups for the variables expressed as frequency was done using the Chi-square test. The comparison between groups of variables related to socio-demographic and anthropometric characteristics, sleep patterns, drowsiness scores, chronotype, SJL, life habits and stress parameters was done using the Student’s t-test for independent samples or the Mann-Whitney Test. In addition, we performed partial Pearson correlation (adjusted for shift) between sleep duration in work days and stress parameters; mean sleep duration and stress parameters; and sleep debt and stress parameters.

The Generalized Linear Model (GzLM) was used to compare the differences of the variables of oxidative stress and antioxidant defense between shifts, adjusted for age and working time per week. The GzLM Test also was used to compare the differences of the variables of oxidative stress and antioxidant defense between shifts, SJL and shift versus SJL interaction, adjusted for age and working time per week. The sequential Šidák test was used to compare estimated marginal means. Multiple regression analysis with the total population, adjusted for age and shift, and the populations of each work shift, adjusted for age and working time per week, was used to determine if the variables of oxidative stress and antioxidant defense were associated with SJL. The statistical analysis of the data was done using the IBM SPSS software version 20.0. Statistical tests with values of *p* < 0.05 were accepted as significant.
